# Family history and obesity in youth, their effect on acylcarnitine/aminoacids metabolomics and non-alcoholic fatty liver disease (NAFLD). Structural equation modeling approach

**DOI:** 10.1371/journal.pone.0193138

**Published:** 2018-02-21

**Authors:** Maria Elena Romero-Ibarguengoitia, Felipe Vadillo-Ortega, Augusto Enrique Caballero, Isabel Ibarra-González, Arturo Herrera-Rosas, María Fabiola Serratos-Canales, Mireya León-Hernández, Antonio González-Chávez, Srinivas Mummidi, Ravindranath Duggirala, Juan Carlos López-Alvarenga

**Affiliations:** 1 Research Divison, Hospital General de México, Mexico City, Mexico; 2 Vinculation Unit Faculty of Medicine UNAM, Instituto Nacional de Medicina Genomica (INMEGEN), Mexico City, Mexico; 3 Harvard Medical School, Boston, Massachusetts, United States of America; 4 Inborn errors of Metabolism Unit, Instituto Nacional de Pediatría, Mexico City, Mexico; 5 Internal Medicine Department, Hospital General de México, Mexico City, Mexico; 6 South Texas Diabetes and Obesity Institute, University of Texas Rio Grande Valley, Edinburg, TX, United States of America; 7 Research department, Universidad Mexico Americana del Norte, Reynosa, Tamaulipas, Mexico; Medical University of Vienna, AUSTRIA

## Abstract

**Background:**

Structural equation modeling (SEM) can help understanding complex functional relationships among obesity, non-alcoholic fatty liver disease (NAFLD), family history of obesity, targeted metabolomics and pro-inflammatory markers. We tested two hypotheses: 1) If obesity precedes an excess of free fatty acids that increase oxidative stress and mitochondrial dysfunction, there would be an increase of serum acylcarnitines, amino acids and cytokines in obese subjects. Acylcarnitines would be related to non-alcoholic fatty disease that will induce insulin resistance. 2) If a positive family history of obesity and type 2 diabetes are the major determinants of the metabolomic profile, there would be higher concentration of amino acids and acylcarnitines in patients with this background that will induce obesity and NAFLD which in turn will induce insulin resistance.

**Methods/Results:**

137 normoglycemic subjects, mean age (SD) of 30.61 (8.6) years divided in three groups: BMI<25 with absence of NAFLD (G1), n = 82; BMI>30 with absence of NAFLD (G2), n = 24; and BMI>30 with NAFLD (G3), n = 31. Family history of obesity (any) was present in 53%. Both models were adjusted in SEM. Family history of obesity predicted obesity but could not predict acylcarnitines and amino acid concentrations (effect size <0.2), but did predict obesity phenotype.

**Conclusion:**

Family history of obesity is the major predictor of obesity, and the metabolic abnormalities on amino acids, acylcarnitines, inflammation, insulin resistance, and NAFLD.

## Introduction

Nearly a third of the world’s population is either obese or overweight [1,2]. Mexico ranks second worldwide in prevalence of combined overweight and obesity in adults at 71.3% [3–5]. Long-term weight loss maintenance is still a great challenge despite advances in the modalities to treat obesity [6].

The classic paradigm proposes obesity as imbalance of positive energy intake, which results in expansion of adipose tissue, and subsequent proinflammatory process due to release of cytokines, C reactive protein (CRP), interferon gamma (IFN**γ**), Tumor Necrosis Factor alpha (TNFα), among many other molecules; additionally, increase of free fatty acids induces lipotoxicity [7–9]. As a consequence, patients with obesity have a higher risk of insulin resistance (IR), lipid dysmetabolism, Type 2 Diabetes (T2DM), Non-alcoholic Fatty Liver Disease (NAFLD), and other chronic complications [9].

Interestingly, not all patients with obesity develop T2DM or NAFLD, furthermore, patients with high genetic predisposition to obesity can acquire NAFLD more easily with minimal environmental exposure. This suggests either additive or synergistic interactions among multiple risk factors that include environment, lifestyle, genome, epigenome, metabolome, and other key factors that play a role in its pathogenesis [10].

Over the past decade, strong emphasis was placed on identifying genetic risk factors for obesity [11–13]. While these efforts yielded some remarkable results, some recent studies have suggested that the use of clinical family history may be more informative than genotyping single candidate genes because it captures both genetic risk as well as the epigenetic markers that are associated with the life style of parents and to the second degree grandparents [14]. A powerful method to assess such interrelationship between genetics, epigenome and the phenotype is metabolomics, which uses highly sensitive technologies such as mass spectroscopy to analyze low-molecular weight intermediates (< 1 kDa) in biological fluids or tissues such as blood, urine, saliva, etc.[15,16]. Recent studies have documented that obese, insulin resistant, and NAFLD, when compared with normal subjects, have differences in blood metabolite profiles, such as glucose, lipids, acylcarnitines and amino acids [17–25]. While metabolic profiling yields copious data, traditional methods of analyses such as cluster analyses and standard linear models can fail to determine functional relationships between the examined variables [26].

In this study, we computed Structural Equation Models (SEM) [27] to integrate multidimensional data into single framework to unravel their interrelationship in the context obesity and related traits. While SEM have been used to reconstruct phenotype networks in genetics, behavioral, and social science [27,28], to the best of our knowledge, it has not been applied to the metabolomics field. Here we used SEM to address the interrelationships between obesity, NAFLD, different degrees of family history of obesity, targeted metabolomics and pro-inflammatory markers.

Based on the aforementioned concepts, we tested two hypotheses:

1. If obesity precedes an excess of free fatty acids that perhaps increase oxidative stress and mitochondrial dysfunction, there will be increased acylcarnitines (AC) in blood in obese patients. Also, there will be an increase of amino acids (AC) and cytokines (INFL). Acylcarnitines will be related to non-alcoholic fatty disease (NAFLD) that will induce insulin resistance (IR). (Obesity→AC+AA → NAFLD → IR and INFL).
2. If a positive family history of obesity (FHOB) is the major determinant of the metabolomic profile, then those subjects will exhibit higher concentration of AC and AA that will induce obesity and NAFLD, which in turn will derive on IR. In this model, we are proposing that obesity is a consequence of previous alteration of fatty acids and amino acids metabolism (FHOB→High AA and AC → Obesity & NAFLD → IR).

## Material and methods

### Study sample

We conducted a cross-sectional study with 137 consecutive patients aged between 18 to 45 years who were recruited from January to October 2012 at the outpatient clinic, Hospital General de Mexico, Mexico City. Subjects were divided into 3 groups: Group 1 BMI<25 (G1), Group 2 BMI>30 (G2) and, group 3 (BMI>30 with NAFLD) (G3). We had made previous simulations, and a priori anticipated unbalanced sample size groups. Family history of diabetes/obesity was recorded, parental first-degree relative was defined as “Direct”, meanwhile, second-degree relatives were “Indirect”. We excluded patients with pregnancy, those who smoke, or consumed more than 10 grams of alcohol/week, those with clinical history of known hepatotoxic medications, diagnosis of cancer, any acute or chronic infectious disease, hypertension, diabetes, chronic kidney disease or any pathological condition during the general examination or laboratory tests.

The study protocol was approved by the Human Ethics Committee at the Hospital General de México, an informed consent was obtained from all subjects. The investigation was conducted according to the principles expressed in the Declaration of Helsinki.

### Procedure

Subjects were invited to participate, if they accomplished inclusion criteria, and signed the informed consent. During the first visit, we recorded blood pressure, BMI and bioelectric impedance parameters using a Quantum IV–Body Composition Analyzer (RJL Systems, USA). Within a week but on different date, patients were instructed to fast 8 hours before a 8 am oral glucose tolerance test (OGTT) with 75 g of glucose. Baseline blood sample was drawn for for glucose, creatinine, urea (to discharge kidney disease), total cholesterol, HDL cholesterol, LDL cholesterol, triglycerides, alanine aminotransferase (AST), and aspartate aminotransferase (ALT) with an AU480 Chemistry System (Beckman Coulter, USA). During the OGTT, we also measured insulin levels through ELISA with an Abnova™ Kit using the V1.24 device (Multiskan Ascent, USA) with intra and inter-assay coefficients of variation (CV) ranging between 1.8 to 2.9%. The Matsuda insulin sensitivity index was calculated as described elsewhere (35). Inflammatory markers measured in blood were IL-6 and TNFα using ELISA with Bioplex-Pro™ Cytokine assays and the Bio-Plex Pro II wash station with a magnetic plate carrier (Bio Rad, USA), CV:4–19%. C Reactive protein was measured with an immunoenzyme assay using microplates (Monobind Inc, USA).

Plasma samples from fasting subjects were used to determine the profiles of 31 endogenous acylcarnitines and 7 amino acids were using Quattro Micro API (MicroMass) tandem mass spectrometer (MS-MS). All procedures for sample preparation and MS-MS analysis were performed by NeoBase no derivatized kit (PerkinElmer, USA) according to the manufacturer's protocol. Briefly, plasma was dried in filter papers and single disks were punched from each spot using a 3 mm punch. One disk was used per well. Using a multichannel pipette, 190 μL of extraction solution containing a mixture of the respective stable isotope-labeled internal standards, was added to each well. The plate was covered with aluminum foil, shaken at 650 x g and incubated for 30 min at 30°C. The plate was finally placed in the auto-sampler for the analysis. Finally, we performed a hepatic ultrasound with the Voluson Pro VTM ultrasound system (GE, USA) with a 3.5MHz transductor. Hepatic ultrasounds have a sensitivity and specificity for NAFLD detection of 80 to 90% (36). Presence of NAFLD was determined if 3 parameters were present: 1) high hepatic echotexture, 2) high attenuation, 3) low portal and hepatic vein visualization.

### Statistical analysis

We described and contrasted demographic, serum biochemical values for the three described groups (Method section); those variables with skewed distributions were normalized using log_10_ transformation. Contrasts by group were computed using Chi-square or one way-ANOVA with Fisher post hoc tests according to variable dimension. For metabolic and inflammatory markers, we computed False discovery Rate (FDR) with the Benjamini–Hochberg procedure [29].

We used Partial Least Squares Discriminant Analysis (PLS-DA) to visually discriminate metabolites and inflammatory markers between the 3 groups. The quality of PLS-DA was assessed using 3 different parameters: R2, Q2 and accuracy. The goodness of fit was quantified by R2 and the predictive ability was indicated by Q2.

To assess the significance of class discrimination a permutation test was conducted where the model was run 1000 times. Variable Importance in Projection (VIP) was calculated as a weighted sum of squares of the PLS loadings taking into account the amount of explained Y-variation.

In order to test the two described hypotheses we used SEM, a generalization of both regression and factor analysis. The rationale for using SEM is that the covariance matrix of the observed variables is a function of a set of parameters that were defined a *priori*. If the model is correct and the parameters are known, then the population covariance matrix would be exactly reproduced by SEM (except for sampling variation). The hypothetical relation among the variables in our models was built based on the conjunction of previous reported data from the literature validated by simulation models that were generated in advance and modified according to modification indexes. To avoid hormonal effects and physiological changes from adolescence and senescence we only included patients between 18 to 45 years old, yet to conserve the required illness diversity for analysis of biological variance. Age did not have any rol as observed neither latent variable for SEM.

The general SEM model can be decomposed into two sub models: a measurement and a structural model. By convention, when graphically representing the model the observed variables are enclosed by rectangles or squares and latent variables are enclosed by ovals or circles. Residuals are always unobserved variables (latent factors) and are represented by ovals or circles. In this study, the *root mean square error of approximation* (RMSEA) was used to evaluate the goodness-of-fit of any model, where a value <0.9 was considered acceptable for our models. Standardized β values >0.2 were relevant in the pathophysiology of our models [27,30–32].

We considered sample size according to the minimum number of patients needed to have good cohesion of factors in our models. For this we did a priori Monte Carlo simulations modeling with beta distribution considering parameters α = 1 and β = 7, to increase the skewness of simulated data. Maximum and minimum values were obtained considering 3 standard deviations of the mean of values obtained from previous studies. We expected at least 20% difference on the effect size of the concentration of amino acids between lean patients and patients with obesity. We also considered an effect size of at least 20% in acylcarnitine concentrations between patients with and without NAFLD. With 137 patients, we had good adjustment of our models that included effect size between 19 and 100%.

All statistical analyses were done using SPSS 18.0, Amos and Metaboanalyst 3.0[33].

## Results

We included 137 normoglycemic subjects, with a mean age of 30.61 (SD 8.6) years (Table 1), seventy percent were women. Group 1 (BMI<25) had 82 subjects; Group 2 (BMI >30) and Group 3 (BMI>30 and NAFLD) group had 24 and 31 subjects, respectively. Family history of obesity (any) was present in 53% of the total population, while family history of diabetes was present in 66%. Table 1 shows mean values of clinical, inflammatory and metabolomic parameters of the 3 groups and Table 2 shows the different degrees of family history of obesity and diabetes.

**Table 1:** Clinical and metabolomic parameters of patients according to 3 groups. Mean (95% CI).

	G1 BMI<25 Mean (95% CI) number = 82	G2 BMI>30; NAFLD (-) Mean (95% CI) number = 24		G3 BMI>30; NAFLD (+) Mean (95% CI) number = 31	p-value	FDR	Factor in SEM Model
**Antropometric**							
Age	28(26,29)^b^	27(24,32)^b^		36(33,39) ^a^	<0.001	NA	
Gender (% Females)	65	87		71	0.098	NA	
BMI	22.2 (22,23)^c^	32.9 (32,34)^b^		36.2 (34,38)^a^	<0.001	NA	Obesity
Abdominal Circumference (cm)	73.42(72,75)^c^	85 (75,95)^b^		102.48 (96,107)^a^	<0.001	NA	Obesity
%FAT	29.7 (28,31)^b^	40.88 (37,45)^a^		41.63 (32,36)^a^	<0.001	NA	Obesity
**Insulin Sensibility**							
Basal Glucose (mg/dl)	86 (85,88)^b^	88 (86,90)^a,b^		91 (89,93)^a^	0.003	0.009	
Glucose 30 min (mg/dl)	123 (118,128)^b^	126 (113,140)^a,b^		142 (134,150)^a^	0.004	0.01	
Glucose 60 min (mg/dl)	109 (102,116)^b^	118 (105,132)^a,b^		135(123,147)a	0.002	0.007	
Glucose 90 min (mg/dl)	100 (96,106)^b^	114 (103,126)^a^		128 (120,136)^a^	<0.001	<0.001	
Glucose 120min (mg/dl)	99 (94,103)	104(101,108)^a,b^		116 (110,126)^a^	0.001	0.002	
Matsuda Index	6.1 (5.4,6.9)^a^	4.8 (4,6)^a^		3.1 (2.4,3.9)^b^	<0.001	<0.001	Matsuda Index
**Lipids and Liver Enzymes**							
Total Cholesterol (mg/dl)	170 (164,176)^b^		173(161,186)^b^	190 (178,202)^a^	0.008	0.02	
Triglycerids (mg/dl)	93 (85,101)^b^		114 (98,132)^b^	170 (141,206)^a^	<0.001	<0.001	
HDL- Cholesterol (mg/dl)	48 (46,51)^a^		44 (39,50)^a,b^	49 (36,42)^b^	0.001	0.002	
LDL- Cholesterol (mg/dl)	100 (95,105)^b^		110 (102,120,)^a,b^	119 (110,129)^a^	0.001	0.003	
ALT (U/L)	19 (17,20)^c^		21 (16,26)^b^	31 (26,37)^a^	<0.001	<0.001	Fatty Liver
AST (U/L)	21 (20,23) ^b^		22 (19,25)^a,b^	26 (23.29)^a^	0.005	0.014	Fatty Liver
**Inflammatory markers**							
CRP(mg/dl)	1.9 (1.7,2.2)^b^	4.2 (3.1,5.7)^a^		4.8 (3.9,5.9)^a^	<0.001	<0.001	CRP
IL-6 (pg/dl)	1.15 (0.96,1.37)	0.66 (0.33,1.3)		0.85 (0.68,1.02)	0.056	0.488	INFL
TNF**α** (pg/dl)	1.14 (0.9,1.43)	75 (0.41,1.3)		0.9 (0.72,1.3)	0.234	0.52	INFL
**Amino acids**							
Arginine (μM)	36.3 (33,40)^b^	42.3(37,48.3)^a,b^		45.2(39.2,52.1)^a^	0.027	0.064	AA1
Citruline (μM)	9 (8.5, 9.5)	9.5 (8.5,10.6)		9.2(8.3,10.35)	0.656	0.787	AA2
Glycine (μM)	101.8 (96.4,105.2)	101.8 (92.5,112)		99.6(92.4,107.3)	0.926	0.936	AA1
Alanine (μM)	112 (106.9,117.6)^b^	122.1(113.6,131.7)^a,b^		128.3 (119.1,138.1)^a^	0.006	0.016	AA2
Leucine (μM)	54.2 (50.6,57.9)^b^	59.3 (54.4, 64.7)^a,b^		63.6(57.9,69.9)^a^	0.022	0.052	AA1
Methionine (μM)	4.5 (4.3,4.8)	4.6(4.29,5.1)		4.7 (4.3,5.12)	0.737	0.804	AA2
Phenylalanine (μM)	20.9 (19.4, 22.4)^b^	24.48 (19.4,22.4)^a,b^		25 (22.7, 27.6)^a^	0.005	0.014	AA1
Tyrosine (μM)	23.2 (22, 24.4)b	26.4 (24,29)^a^		28 (26.6,31.2)^a^	<0.001	<0.001	AA2
Valine (μM)	56.6 (53.8,59.5)b	63.035 (12.9)^a,b^		67.69 (16.2)^a^	0.004	0.011	AA1
Ornitine (μM)	7.9 (7.4,8.6)b	10.15 (8.7,11.8)^a^		10.23(9.1,11.4)^a^	<0.001	0.001	AA2
Proline (μM)	61.2(57.1,65.7)a	64.6(56.8,73.6)^a,b^		74.5(67.19,82.79)^a^	0.012	0.031	AA2
**Acylcarnitines**							
C0 (μM)	12.4 (11.7,13.2)^b^	13.9 (12.8,15.3)^a^		13.8(12.6,15.2)^a^	0.044	0.09	AC3
C2 (μM)	0.12 (0.11,0.13)	0.13(0.10,0.15)		0.13 (0.10,0.16)	0.794	0.875	AC1
C3 (μM)	0.029 (0.026,0.031)	0.033(0.028,0.04)		0.034(0.029,0.040)	0.075	0.37	AC1
C4 (μM)	0.035 (0.033,0.036)	0.034(0.030,0.039)		0.035(0.032,0.039)	0.922	0.922	AC1
C5 (μM)	0.030 (0.027,0.032)	0.035(0.031,0.039)		0.035(0.028,0.039)	0.078	0.39	AC2
C6 (μM)	0.016 (0.0148,0.173)	0.017(0.015,0.02)		0.017(0.015,0.02)	0.243	0.36	AC2
C8 (μM)	0.036(0.033, 0.04)	0.039(0.034,0.04)		0.036(0.03,0.04)	0.745	0.745	AC2
C10 (μM)	0.07 (0.06, 0.07)	0.069(0.06, 0.07)		0.06 (0.05, 0.07)	0.11	0.22	AC2
C10:1 (μM)	0.105 (0.10,0.11)	0.10 (0.0.11, 0.98)		0.103 (0.09,0.11)	0.571	0.6852	AC2
C10:2 (μM)	0.018 (0.0.017,0.019)^b^	0.018(0.015,0.021)^a,b^		0.021(0.019,0.023)^a^	0.05	0.08	AC2
C12 (μM)	0.38 (0.036,0.041)	0.037(0.034,0.042)		0.036(0.032,0.040)	0.353	0.45	AC2
C12:1 (μM)	0.049(0.047, 0.052)	0.051(0.048,0.055)		0.047(0.043,0.052)	0.769	0.877	AC2
C14(μM)	0.014 (0.013,0.015)	0.014 (0.012, 0.016)		0.015(0.013, 0.017)	0.887	0.877	AC2
C14:1 (μM)	0.037 (0.035,0.039)	0.037(0.033,0.041)		0.036(0.032,0.040)	0.037	0.22	AC2
C14:2 (μM)	0.015 (0.014,0.016)	0.015 (0.013,0.018)		0.014(0.012,0.017)	0.579	0.579	AC2
C16 (μM)	0.029 (0.028, 0.031)	0.034(0.030, 0.038)		0.031 (0.027, 0.034)	0.859	0.859	AC3
C16:1 (μM)	0.009 (0.007, 0.011)	0.008(0.004, 0.015)		0.01 (0.009, 0.011)	0.813	0.89	AC3
C18:1OH (μM)	0.0005(0.0002, 0.0012)	0.0007 (0.0001,0.003)		0.0008(0.0002, 0.002)	0.83	0.859	AC4
C18:2 (μM)	0.015 (0.014, 0.016)	0.018(0.016, 0.020)		0.016(0.013, 0.018)	0.12	0.24	AC3
C18OH (μM)	0.001 (0.0009,0.0004)	0.0005(0.0001, 0.002)		0.0001(0.00005, 0.0006)	0.377	0.37	AC4

Contrast between groups by one-way ANOVA test. Fisher Post hoc test was done. Variable values were recovered using antilogarithms. FDR = False Discovery Rate. Only the acylcarnitines and amino acids used in SEM are showed in the table. Superscript letters show homogenous groups.

**Table 2:** Family history of obesity and diabetes in 3 groups.

		G1 (%)	G2 (%)	G3 (%)	p-value	Variable names in SEM Model
	number = 137	number = 82	number = 24	number = 31		
**Family History of Obesity (FHO)**	72 (53)	38 (46)	14 (58)	20 (65)	0.14	
Direct FHO	46 (34)	23 (28)	14 (58)	9 (29)	0.02	
Mother FHO	32 (23)	14(17)	13 (54)	5 (16)	<0.001	MomFHO
Father FHO	24 (17)	15 (60)	6 (25)	5 (16)	0.57	DadFHOB
Indirect FHO	54 (39)	30 (37)	10 (41.7)	14 (45)	0.57	IndFHOB

Family history of obesity in G1, G2, G3. G1 = BMI<25, NAFLD negative; G2 = BMI>30, NAFLD negative; G3 = BMI>30+NAFLD. Chi square test.

### Clinical, metabolomic and inflammatory differences between G1-G3

Clinical differences were observed among the three groups as expected: the patients with obesity with or without NAFLD (G2, G3) had higher BMI, percentage of fat and abdominal circumference (p<0.001) (Table 1). There were no differences in exercise activity between groups (p>0.05).

Even though none of the patients had diabetes or glucose intolerance, both G2 and G3 had higher glucose and insulin levels with lower Matsuda index (p<0.001). These groups also had higher cholesterol and triglyceride levels (p<0.001) (Table 1); and G3 only had high levels of CRP, arginine, alanine, leucine, phenylalanine, tyrosine, valine, ornithine, and proline (p<0.01).

Medium and long chain acylcarnitines such as C8:1, C10:2 and C18:1 had higher serum levels (p<0.05) in patients with obesity regardless of NAFLD compared to lean controls, though no differences were found between obese patients with and without NAFLD.

**Fig 1:**
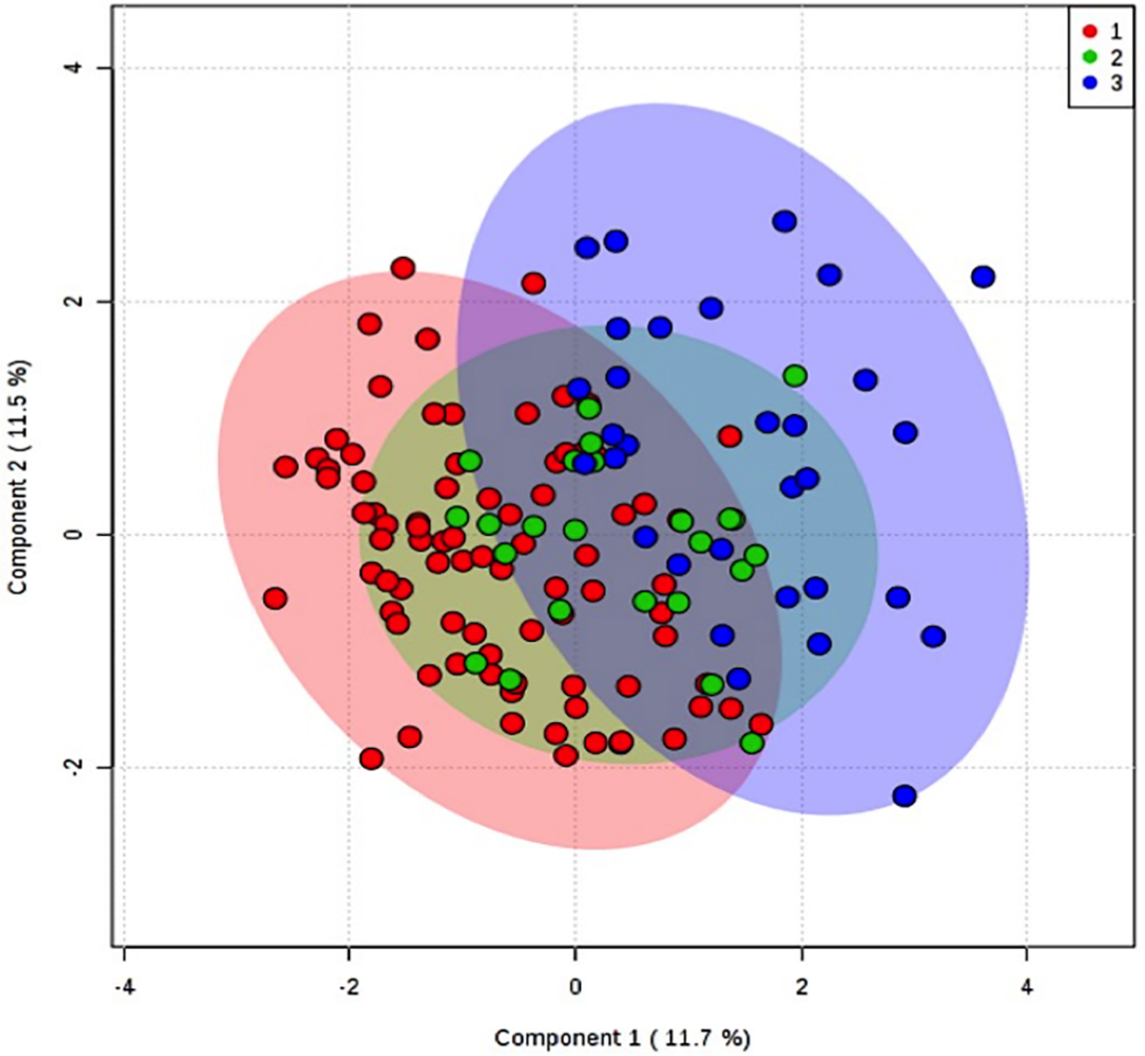
PLS-DA of metabolytes in 3 groups. 1 = BMI<25, 2 = BMI<30 and NAFLD negative, and 3 = BMI>30 and NAFLD positive.

PLS-DA analysis demonstrated a mild separation between G1 and G3, while as expected G2, was over imposed between G1 and G3; three components explained 62% of the variance (accuracy 0.68, R2 0.6, Q2 0.39, p<0.001). VIP scores > 1.2 were: CRP, insulin, triglycerides, C:16: 1OH, C8:1, ornithine and tyrosine (Fig 1).

### Structural equation models

In order to test our hypothesis, we created two structural equation models and a third derived from the other two. An exploratory factor analysis clustered acylcarnitines into 4 factors and amino acids into two factors. These models are shown in Table 1. We only used TNF**α** and IL-6 (both grouped as “INFL”) and CRP in our models since they are the most common inflammatory makers reported in the literature.

**Fig 2:**
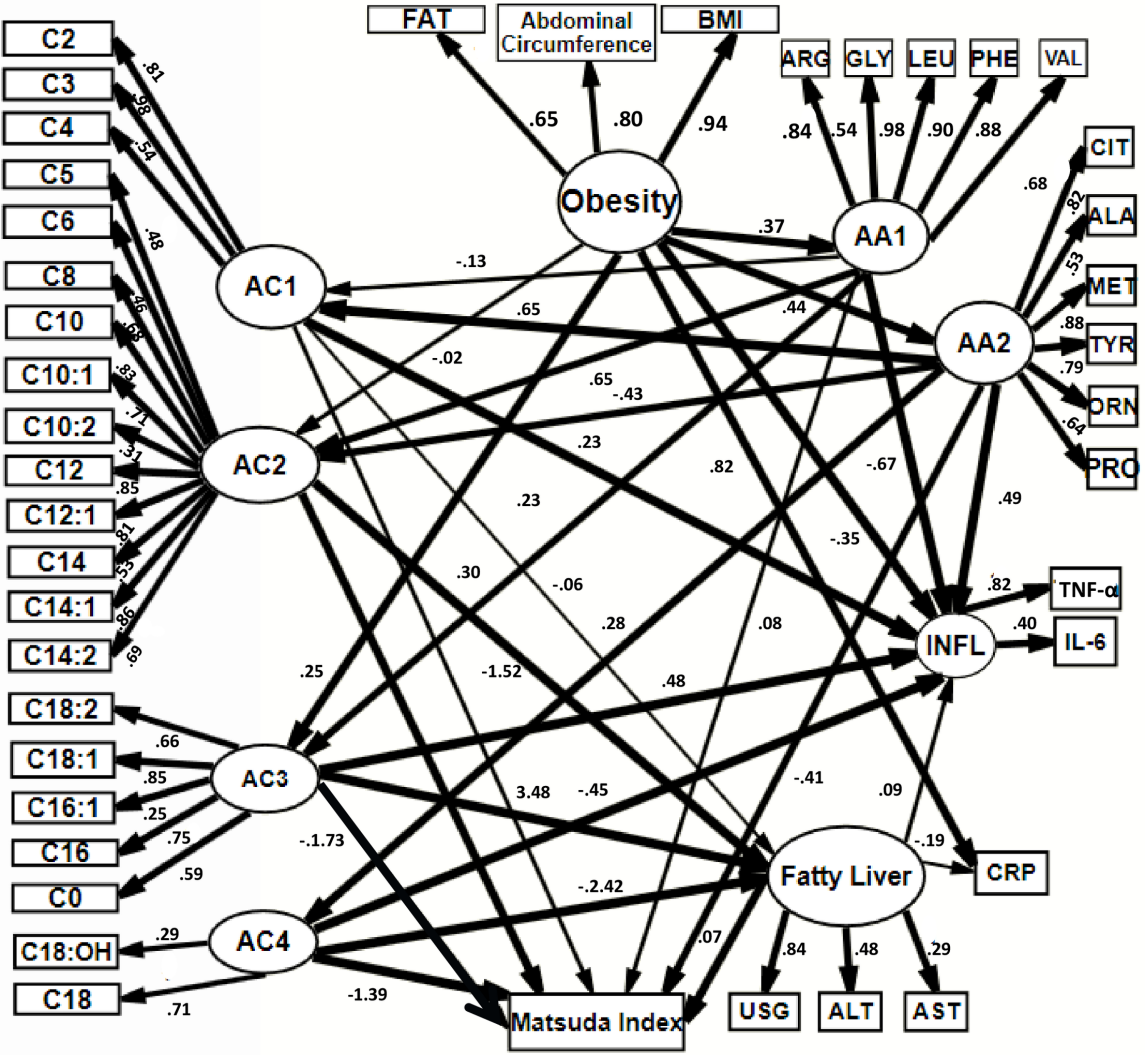
SEM hypothesis 1. Obesity correlates with plasmatic amino acids, which contributes to increase of specific acylcarnitines and inflammatory markers. Obesity correlated directly with acylcarnitines. These were associated with NAFLD and then related to inflammatory markers and insulin resistance. Circles = latent variables, rectangles = observed variables, e = error term. Latent variable obesity is formed by: BMI = Body mass Index, Abd_circumf = abdominal circumference, FAT = % of Fat. AA1 and AA2 = latent variables that represent factor for amino acids AC1, AC2, AC3 and AC4 = latent variables that represent factors grouped for acylcarnitines. C:0,C2-C18:2. ALA = alanine, CIT = citrulline, Met = methionine, TYR = tyrosine, ORN = ornithine, PRO = proline, ARG = arginine, GLY = glycine, LEU = leucine, PHE = phenylalanine, VAL = valine. CRP = C reactive protein. TNF**α** = Tumor necrosis factor alpha, IL-6 = Interleukine-6, both form latent variable INFL = inflammatory markers. Latent variable NAFLD integrates USG = liver ultrasound. ALT = Alanine aminotransferase, AST = Aspartate aminotransferase. Lines in bold correspond to standardized β estimates > 0.2. Numbers in each line correspond to standardized β estimate. To simplify, error terms were not included in the figure.

The first model (Fig 2, S1 Table) showed that obesity correlates with plasma amino acids, which contributes to increase of specific acylcarnitins and inflammatory markers. The excess free fatty acids were associated with NAFLD and also with inflammatory markers and insulin resistance in that order. The RMSEA was 0.078 (0.0.71, 0.084), and standardized β values > 0.2 (shown in parenthesis) supported obesity as predictor of endogenous variables such as amino acid concentration grouped in both factors AA1 and AA2 (0.37 and 0.44 respectively). Amino acids grouped in the factor AA1 predicted the blood concentration of medium, long and very long chain acylcarnitines grouped in AC2- AC4 (0.65 and 0.30). AA2 predicted AC1, AC2 and AC4 (0.65, -0.43 and 0.28). Short, long and very long chain acylcarnitines, AC1, AC3, and AC4, predicted inflammatory markers TNF**α** and IL-6 (INFL) (0.23, 0.48, -0.45), while amino acids grouped in AA1 predicted negatively INFL (-0.67), and amino acids grouped in AA2 predicted positively (0.49). AC2-AC4 was associated with NAFLD (-1.52, 3.48, -2.42). Finally, AC3, AC4, and AA2 predicted Matsuda index (1.39, 0.72, -0.41).

**Fig 3:**
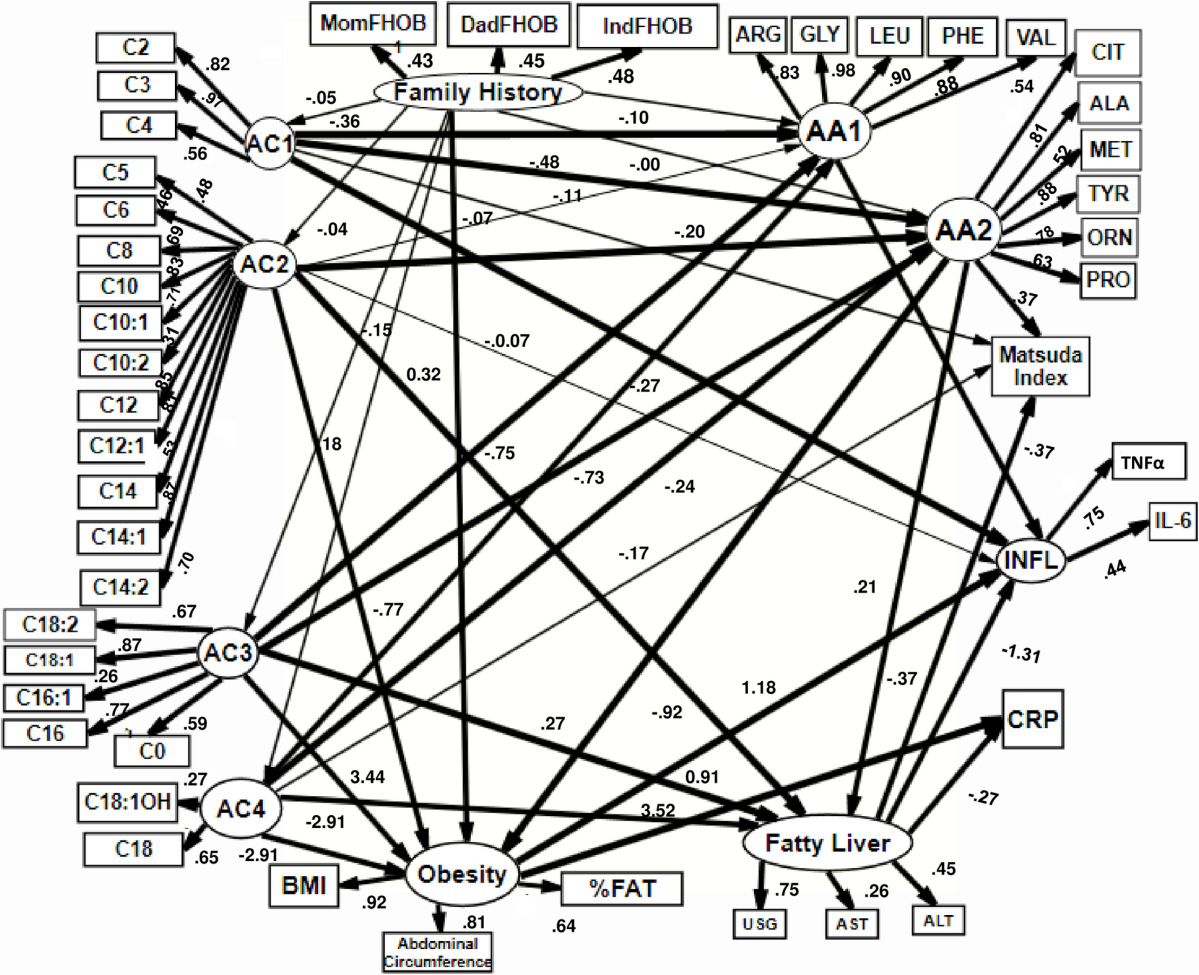
SEM hypothesis 2. Positive family history of obesity was the mayor determinant of acylcarnitines and amino acids, This were associated with NAFLD, obesity, insulin resistance and pro-inflammatory process. In this model we proposed obesity as a secondary, or a consequence of a previous alteration of fatty acid and amino acids metabolism. Circles = latent variables, rectangles = observed variables, e = error term. Family History of obesity is formed by: IndFOB = Second degree family history of obesity. DadFHOB = Parental history of obesity. MomFHOB = Maternal family history of Obesity. Latent variable obesity is formed by: BMI = Body mass Index, Abd_circumf = abdominal circumference, FAT = % of Fat. AA1 and AA2 = latent variables that represent factor for amino acids AC1, AC2, AC3 and AC4 = latent variables that represent factors grouped for acylcarnitines. C:0,C2-C18:2. ALA = alanine, CIT = citrulline, Met = methionine, TYR = tyrosine, ORN = ornithine, PRO = proline, ARG = arginine, GLY = glycine, LEU = leucine, PHE = phenylalanine, VAL = valine. CRP = C reactive protein. TNF**α** = Tumor necrosis factor alpha, IL-6 = Interleukine-6, both form latent variable INFL = inflammatory markers. Latent variable NAFLD integrates USG = liver ultrasound. ALT = Alanine aminotransferase, AST = Aspartate aminotransferase,. Lines in bold correspond to standardized β estimates > 0.2. Numbers in each line corresponds to standardized β estimate Lines in bold correspond to standarized B estimates > 0.2. Numbers in line correspond to standardized β estimates. To simplify, error terms were not included in the figure.

In the second model (Fig 3, S2 Table), a positive family history of obesity (exogen variable), was the major determinant of acylcarnitines and amino acids. This was associated with NAFLD, obesity, insulin resistance and pro-inflammatory process. In this model, we proposed obesity as a secondary, or a consequence of a previous alteration of fatty acid and amino acids metabolism. The RMSEA of this model was 0.075 (0.069, 0.081).

Standardized β estimates for family history of obesity to predict acylcarnitines and amino acids were < 0.2. However, the β estimate for family history of obesity to predict obesity was 0.32 (see Fig 3).

**Fig 4:**
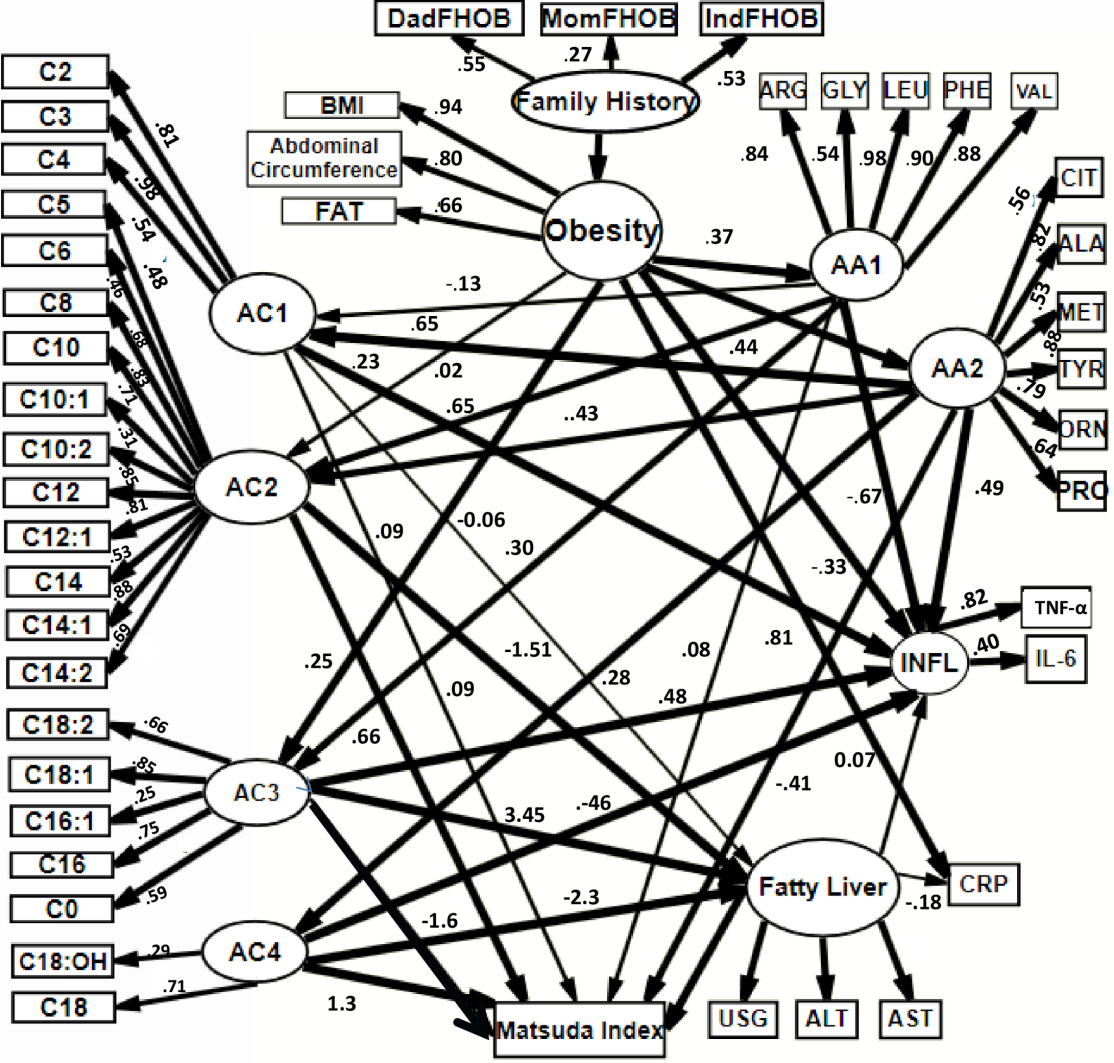
SEM model 3. Family history predicted obesity. This correlates with plasmatic amino acids that contribute to an increase of specific acylcarnitins and inflammatory markers. This was associated with NAFLD and then related to inflammatory markers and insulin resistance. Circles = latent variables, rectangles = observed variables, e = error term. Family History of obesity is formed by: IndFOB = Second degree family history of obesity. DadFHOB = Parental history of obesity. MomFHOB = Maternal family history of Obesity. Latent variable obesity is formed by: BMI = Body mass Index, Abd_circumf = abdominal circumference, FAT = % of Fat. AA1 and AA2 = latent variables that represent factor for amino acids AC1, AC2, AC3 and AC4 = latent variables that represent factors grouped for acylcarnitines. C:0,C2-C18:2. ALA = alanine, CIT = citrulline, Met = methionine, TYR = tyrosine, ORN = ornithine, PRO = proline, ARG = arginine, GLY = glycine, LEU = leucine, PHE = phenylalanine, VAL = valine. CRP = C reactive protein. TNF**α** = Tumor necrosis factor alpha, IL-6 = Interleukine-6, both form latent variable INFL = inflammatory markers. Latent variable NAFLD integrates USG = liver ultrasound. ALT = Alanine aminotransferase, AST = Aspartate aminotransferase. Lines in bold correspond to standardized β estimates > 0.2. Numbers in each line corresponds to standardized β estimate. To simplify, error terms were not included in the figure.

Based on the results of the previous models we created a third model were family history predicted obesity. This correlated with plasma amino acids that contributed to an increase of specific acylcarnitins and inflammatory markers. The excess of free fatty acids related to obesity was associated with NAFLD and then related to inflammatory markers and insulin resistance. The RMSEA of the model was 0.075 (0.069, 0.081) (Fig 4, S3 Table).

## Discussion

The presented SEM analysis evaluated hypothetical causal relationships of phenotypic, metabolomics, inflammatory markers and family history of obesity in an integrated model and found that the family history strongly correlates with a subject’s obesity.

Family history of obesity serves as a proxy for an individual’s genetic, epigenetic and fetal development background and obesity results in severe disruption of regulation of key metabolic enzymes and pathways as indicated by acylcarnitines and amino acids, and both metabolites predict inflammation, insulin resistance, obesity and NAFLD.

Traditional statistical methods such as ANOVA, Chi-square and PLS-DA demonstrated demographic, clinic and metabolomics showed differences among G1-G3 groups, but provided insufficient information about their relationship, also multiple ANOVA increase the risk of type 1 error. Our use of SEMs overcame such drawbacks and provided the following advantages: (1) good cohesion between variables; (2) small error values that led to clean sample selection; (3) good effect size (4) biological support, and (5) detection of previously undetected correlations.

The first model we proposed obesity results from imbalance in positive energy intake, giving adipose tissue expansion. There is an increase of free fatty acids and a disruption in mitochondrial β oxidation that results in an increase of acylcarnitines. An increase of amino acids also predicts increased alfa-ketoacids with subsequent increase in acylcarnitins, specifically short chain. This metabolic disruption predicts a inflammatory process, NAFLD and insulin resistance.

It has been previously published that branched-chain amino acid–derived C3- and C5-carnitine, with fatty acids derived C6 and C8 acylcarnitines have high plasma concentration in patients with obesity and T2D compared with lean controls [19].

Our models support every amino acids biochemical structure is related to obesity and not only branched chained amino acids (BCAA); but only amino acids grouped in Factor AA2 predicted Matsuda index. A study conducted in India and China showed a correlation of HOMA with alanine, proline, valine, and leucine [34]. It has been previously reported that the increased concentrations of BCAA associated with IR is related to chronic phosphorylation of the mammalian target of rapamycin, c-jun N-terminal kinase and insulin receptor substrate 1[19]. Also, there are decreased insulin-stimulated tyrosine phosphorylation of IRS-1 and IRS-2; decreased binding of grb2 and the p85 subunit of phosphatidylinositol 3-kinase to IRS-1 and IRS-2, and a marked inhibition of insulin-stimulated phosphatidylinositol 3-kinase[35]. However, it still remains unclear the mechanisms underlying the non-BCAA association with obesity and IR.

Niu *et al*. has previously studied the relation between amino acids and pro-inflammatory response. They reported histidine and arginine were negatively associated to IL-6 and CRP in obese women [36]. We found similar findings in our model. In another study, C57BL/6J mice were fed with high fat diet and compared with lean controls, mRNA levels in down regulation genes associated to branched-chain amino acid pathways in visceral adipose showed a decrease in the metabolism of BCAA/TCA cycle related to increased concentrations of TNFα, IL-6, IL-1β, and IFNγ [37]. Our models supported a clear interrelationship between all analyzed amino acid residues with IL-6, TNF**α** and CRP.

Obesity is associated with high concentration of short chain acylcarnitines, which may reflect higher lipid fluxes, mitochondrial and β-oxidation overload, incomplete channeling of fatty acids (FA) to complete oxidation, or the oxidation rate of amino acids. There is a lack of clarity if short chain acylcarnitines have a negative effect on insulin signaling processes and if this effect is rather indirect. Our models did not support significant relationship (standardized β values < 0.2) between short chain acylcarnitines and Matsuda index [38].

Schooneman *et al*., reported patients with obesity have lower carnitine palmitoyltransferase 1 (CPT1) and citrate synthase content that promote lower fatty acid oxidation and an increase in long chain acylcarnitines [39]. The latter have been associated with insulin resistance, making a role for long-chain acylcarnitines conceivable in this mechanism [40]. Our models defined a clear correlation between long chain acylcarnitines, obesity and Matsuda index.

It has been reported previously that patients with NAFLD have higher levels of free carnitine, and short chain acylcarnitines such as C3-C5 [23] and long chain acylcarnitines such as C18, C18:2, C16. [24]. A mouse model reported an association of C:10 acylcarnitine and NAFLD [17]. In our study, free carnitine was higher in patients with NAFLD and medium and long acylcarnitines concentrations negatively predicted presence of NAFLD. The normal AST and ALT reference values in Mexican population are 23.7 ± 6.3 IU/L and 20.3 ± 7.6 IU/L respectively. [41] The G3 had ALT levels discreetly higher in our group. High ALT serum concentration correlate with esteatohepatitis. The difference in metabolomics of acylcarnitins and amino acids in esteatohepatitis vs steatosis has been explored previosuly by Satish C. Kalhan *et al*.[23] they obtained biopsies from patients with steatosis and steatohepatitis. They did not find any difference between groups. In an other study conducted by J *Barr et al*. (39), there were differences in acylcarnitines in obese class III, but not in lean subjects, neither obese classes I and II, suggesting that the differences were related to obesity not by inflammatory process of the liver.

Studies have suggested that acylcarnitines may be involved in inflammation. A study using mouse bone marrow derived macrophages treated with L-C12-carnitine polarized towards the M1 pro-inflammatory phenotype, down regulating AMPK and secreted pro-inflammatory cytokines such as IL-3, IL-6, IL-12, IL-11, IL-16, IL-23 y TNFα [42].

In a study using a murine macrophage RAW 264.7 cell line, C12 and C14 acylcarnitines significantly stimulated nuclear factor kappa-B activity (up to 200% of controls) in RAW264.7 cells [43,44].

Our model shows for the first time that short chain and long chain acylcarnitines may correlate with IL-6 and TNF-α, experimental models are need to prove if short chains C2 to C4 acylcarnitines predict inflammatory markers directly, or they have an indirect effect threw amino acids pathways. New models must be done to study the relation between inflammation, acylcarnitines and NAFLD which relations seem not necessary proportional to BMI.

Our second model defined family history explains different capability for the metabolism of amino acids, and mitochondrial β oxidation leading to increase the circulating levels who could lead to the development of obesity, IR and NAFLD. However, the B estimates from this model have very low effect. Other type of study design must be made in the future to test this hypothesis; whole genomic sequencing could be the starting point.

Finally, the novelty of the results shows that family history of obesity does predict obesity phenotype, with a standardized β estimate of 0.3. Once obesity phenotype has established acylcarnitne and aminoacide disruption was supported.

We did not included a group of BMI<25 with NAFLD since their physiopathology seems to be independent of obesity and insulin resistance. This group deserve a more complete study in the future. Some mechanisms proposed are related to genetic alteration in transportation of triglycerides and cholesterol in the body, such as mutations in cholesteryl ester transfer protein, sterol regulatory element binding protein or apolipoprotein 3. The widely studied PNPLA3 gene, encoding for adiponutrin, is an example of a genetic mutation independent of obesity and insulin resistance case. The most relevant polymorphism, I148M is associated with a decreased lipolytic activity. It is also associated with higher aminotransferases levels, but not with insulin resistance. In a previous published paper we had a group of patients with BMI<25 and NAFLD which showed the higher inflammatory process. So if we had included this fourth group in our present models we probably would found higher alteration in β oxidation and inflammatory markers[45–47].

Our study has certain limitations. We evaluated family history as a nominal (dichotomous) variable, which could produce mild instability in the precision of our coefficients, but we concluded this effect was minimal and methodologically tolerable. Our study sample had more women than men, which is in accordance with attendance to the local hospital. In addition, ultrasonography is not considered as the gold standard for NAFLD diagnosis, however this is a screening method with high sensitivity/specificity accuracy.

Another limitation is that we cannot conduct biopsies in this population for ethical reasons. In addition, due to the study design, we were not able to obtain longitudinal data metabolomic profile. However, SEM does let us evaluate dose–response of the variables interrelationships.

It would be interesting to apply SEM based approaches on larger scale studies with other family history approach, and omics area for example proteomics, epigenomics transcriptomics, etc.

## Conclusion

Our study provides SEM that support that family History of obesity, correlates with patients obesity which results in several disruption in the regulation of key metabolic enzymes and pathways that predicts metabolomics (acylcarnitines and amino acids) and this predicts inflammation, insulin resistance, obesity and NAFLD.

## Supporting information

S1 Table Model 1, first hypothesis. Standardized and not standardized β values (β = not standardized estimate, Std β = standardized estimate). BMI = Body Mass Index. Abd_circumf = abdominal circumference, FAT = % of Fat. AC1, AC2, AC3 and AC4 = factors grouped for acylcarnitines C2-C18:2. AA1 and AA2 factors grouped for amino acids. ALA = alanine, CIT = citrulline, Met = methionine, TYR = tyrosine, ORN = ornithine, PRO = proline, ARG = arginine, GLY = glycine, LEU = leucine, PHE = phenylalanine, VAL = valine. CRP = C reactive protein. INFL = inflammatory markers, TNF-a = Tumor necrosis factor alpha, IL-6 = Interleukine 6. USG: liver ultrasound. ALT = Alanine aminotransferase, AST = Aspartate aminotransferase.
(PDF)

S2 Table Model 2, second hypothesis. Standardized and not standardized β values (β = not standardized estimate, Std β = standardized estimate). AC1, AC2, AC3 and C4 = factors grouped for acylcarnitines C2-C18:2. AA1 and AA2 factors grouped for amino acids. ALA = alanine, CIT = citrulline, Met = methionine, TYR = tyrosine, ORN = ornithine, PRO = proline, ARG = arginine, GLY = glycine, LEU = leucine, PHE = phenylalanine, VAL = valine. BMI = Body mass Index. Abd_circumf = abdominal circumference, FAT = % of Fat. CRP = C reactive protein. INFL = inflammatory markers, TNF-a = Tumor necrosis factor alpha, IL-6 = Interleukine 6. USG: liver ultrasound. ALT = Alanine aminotransferase, AST = Aspartate aminotransferase. DadFHOB = Parental family History of obesity. MomFHOB = Maternal family History of obesity. IndFHOB: Second degree family history of obesity.
(PDF)

S3 Table Model 3. Standardized and not standardized β values (β = not standardized estimate, Std β = standardized estimate). BMI = Body mass Index. Abd_circumf = abdominal circumference, FAT = % of Fat. AC1, AC2, AC3 and AC4 = factors grouped for acylcarnitines C2-C18:2. AA1 and AA2 factors grouped for aminoacids. ALA = alanine, CIT = citrulline, Met = methionine, TYR = tyrosine, ORN = ornithine, PRO = proline, ARG = arginine, GLY = glycine, LEU = leucine, PHE = phenylalanine, VAL = valine. CRP = C reactive protein. INFL = inflammatory markers, TNF-a = Tumor necrosis factor alpha, IL-6 = Interleukine 6. USG: liver ultrasound. ALT = Alanine aminotransferase, AST = Aspartate aminotransferase. IndFOB = Second degree family history of obesity. DadFHOB = Parental history of obesity. MomFHOB = Maternal family hitory of Obesity.
(PDF)
