# Correction: Family history and obesity in youth, their effect on acylcarnitine/aminoacids metabolomics and non-alcoholic fatty liver disease (NAFLD). Structural equation modeling approach

**DOI:** 10.1371/journal.pone.0198379

**Published:** 2018-05-24

**Authors:** Maria Elena Romero-Ibarguengoitia, Felipe Vadillo-Ortega, Augusto Enrique Caballero, Isabel Ibarra-González, Arturo Herrera-Rosas, María Fabiola Serratos-Canales, Mireya León-Hernández, Antonio González-Chávez, Srinivas Mummidi, Ravindranath Duggirala, Juan Carlos López-Alvarenga

There are missing affiliations for the second author. The affiliations should appear as: Felipe Vadillo-Ortega^2,8,9^

**2** Vinculation Unit Faculty of Medicine UNAM, Instituto Nacional de Medicina Genomica (INMEGEN), Mexico City, **8** Perinatology Research Branch, Program for Perinatal Research and Obstetrics, Division of Intramural Research Eunice Kennedy Shriver National Institute of Child Health and Human Development, National Institutes of Health/US. Department of Health and Human Services, Bethesda, MD20892 and Detroit, MI, **9** Department of Obstetrics and Gynecology, Wayne State State University School of Medicine, Detroit, MI.
