# Supplementary material for: Family history and obesity in youth, their effect on acylcarnitine/aminoacids metabolomics and non-alcoholic fatty liver disease (NAFLD). Structural equation modeling approach
Source: PLoS One. 2018 Feb 21;13(2):e0193138. doi: 10.1371/journal.pone.0193138 (PMC5821462; doi:10.1371/journal.pone.0193138)
Supplement: S1 Table — Standardized and not standardized β values (β = not standardized estimate, Std β = standardized estimate). BMI = Body Mass Index. Abd_circumf = abdominal circumference, FAT = % of Fat. AC1, AC2, AC3 and AC4 = factors grouped for acylcarnitines C2-C18:2. AA1 and AA2 factors grouped for amino acids. ALA = alanine, CIT = citrulline, Met = methionine, TYR = tyrosine, ORN = ornithine, PRO = proline, ARG = arginine, GLY = glycine, LEU = leucine, PHE = phenylalanine, VAL = valine. CRP = C reactive protein. INFL = inflammatory markers, TNF-a = Tumor necrosis factor alpha, IL-6 = Interleukine 6. USG: liver ultrasound. ALT = Alanine aminotransferase, AST = Aspartate aminotransferase. (PDF) [file pone.0193138.s001.pdf]

S1 Table. SEM Model 1 estimates.

Factors/variables		β	β std	S.E	p-value
Obesity	AA1	0.705	0.369	0.181	<0.001
	AA2	0.337	0.436	0.087	<0.001
	Abd_circunf	0.886	0.804	0.109	<0.001
	AC2	0.031	0.021	0.14	0.823
	AC3	0.284	0.251	0.091	0.002
	BMI	1.265	0.936	0.142	<0.001
	FAT	1	0.654		
	INFL	-1.661	-0.351	1.576	0.292
	PCR	3.268	0.819	0.848	<0.001
AA1	AC1	-0.065	-0.134	0.091	0.474
	AC2	0.518	0.653	0.135	<0.001
	AC3	0.179	0.301	0.046	<0.001
	ARG	1	0.843		
	GLY	0.312	0.54	0.046	<0.001
	INFL	-1.655	-0.669	0.686	0.016
	LEU	0.798	0.976	0.048	<0.001
	Matsuda Index	0.14	0.082	0.344	0.684
	PHE	0.794	0.902	0.055	<0.001
	VAL	0.58	0.883	0.053	<0.001
AA2	AC1	0.779	0.649	0.275	0.005
	AC2	-0.839	-0.428	0.309	0.007
	AC4	0.512	0.278	0.18	0.004
	ALA	1.242	0.819	0.183	<0.001
	CIT	1	0.558		
	INFL	2.982	0.488	1.917	0.12
	Matsuda Index	-1.719	-0.407	0.992	0.083
	MET	0.816	0.526	0.16	<0.001
	ORN	1.969	0.794	0.294	<0.001
	PRO	1.434	0.643	0.244	<0.001
	TYR	1.54	0.885	0.218	<0.001
AC1	C2	2.471	0.81	0.364	<0.001

	C3	2.324	0.982	0.345	<0.001
	C4	1	0.543		
	Fatty Liver	-0.027	-0.058	0.042	0.514
	INFL	1.174	0.23	0.655	0.073
	Matsuda Index	0.31	0.088	0.428	0.469
AC2	C10	1.264	0.832	0.138	<0.001
	C10:1	0.605	0.71	0.075	<0.001
	C10:2	0.317	0.306	0.092	<0.001
	C12	0.908	0.854	0.097	<0.001
	C12:1	0.671	0.815	0.075	<0.001
	C14	0.694	0.53	0.117	<0.001
	C14:1	0.857	0.864	0.091	<0.001
	C14:2	1	0.695		
	C5	0.593	0.48	0.108	<0.001
	C6	0.557	0.455	0.109	<0.001
	C8	1.075	0.684	0.141	<0.001
	Fatty Liver	-0.442	-1.522	0.201	0.028
	Matsuda Index	1.559	0.723	4.696	0.74
AC3	C0	0.707	0.594	0.102	<0.001
	C16	1	0.753		
	C16:1	1.016	0.248	0.359	0.005
	C18:1	1.267	0.848	0.127	<0.001
	C18:2	1.16	0.656	0.154	<0.001
	Fatty Liver	1.351	3.484	0.553	0.015
	INFLL	1.993	0.478	1.422	0.161
	Matsuda I	-4.987	-1.731	14.397	0.729
AC4	Fatty Liver	-0.747	-2.416	0.325	0.021
	C18	1	0.705		
	C18:1OH	3.704	0.287	1.263	0.003
	INFL	-1.51	-0.454	1.154	0.191
	Matsuda Index	3.187	1.387	8.052	0.692
Fatty Liver	ALT	2.81	0.48	0.637	<0.001
	AST	1	0.289		
	INFL	0.976	0.091	3.685	0.791

	Matsuda Index	0.491	0.066	10.24	0.962
	NAFLD	9.946	0.838	3.272	0.002
	PCR	-1.73	-0.191	1.939	0.372
INFL	TNFa	1	0.822		
	IL-6	0.477	0.396	0.257	0.064
