# Supplementary material for: Family history and obesity in youth, their effect on acylcarnitine/aminoacids metabolomics and non-alcoholic fatty liver disease (NAFLD). Structural equation modeling approach
Source: PLoS One. 2018 Feb 21;13(2):e0193138. doi: 10.1371/journal.pone.0193138 (PMC5821462; doi:10.1371/journal.pone.0193138)
Supplement: S2 Table — Standardized and not standardized β values (β = not standardized estimate, Std β = standardized estimate). AC1, AC2, AC3 and C4 = factors grouped for acylcarnitines C2-C18:2. AA1 and AA2 factors grouped for amino acids. ALA = alanine, CIT = citrulline, Met = methionine, TYR = tyrosine, ORN = ornithine, PRO = proline, ARG = arginine, GLY = glycine, LEU = leucine, PHE = phenylalanine, VAL = valine. BMI = Body mass Index. Abd_circumf = abdominal circumference, FAT = % of Fat. CRP = C reactive protein. INFL = inflammatory markers, TNF-a = Tumor necrosis factor alpha, IL-6 = Interleukine 6. USG: liver ultrasound. ALT = Alanine aminotransferase, AST = Aspartate aminotransferase. DadFHOB = Parental family History of obesity. MomFHOB = Maternal family History of obesity. IndFHOB: Second degree family history of obesity. (PDF) [file pone.0193138.s002.pdf]

**S2 Table. SEM Model 2 estimates**

| Factors/variables | | β | β std | S.E | p-value |
|---|---|---|---|---|---|
| Family History | AC1 | -0.021 | -0.05 | 0.053 | 0.698 |
| | AA1 | -0.084 | -0.104 | 0.094 | 0.369 |
| | AA2 | 0.001 | 0.004 | 0.038 | 0.973 |
| | AC2 | 0.028 | 0.042 | 0.09 | 0.753 |
| | AC3 | 0.077 | 0.15 | 0.074 | 0.3 |
| | AC4 | 0.106 | 0.182 | 0.09 | 0.238 |
| | DadFHOB | 0.949 | 0.453 | 0.345 | 0.006 |
| | IndFHOB | 1.282 | 0.476 | 0.458 | 0.005 |
| | MomFHOB | 1 | 0.429 | | |
| | OB | 0.14 | 0.324 | 0.063 | 0.027 |
| AA1 | INFL | -0.705 | -0.3 | 0.368 | 0.055 |
| | ARG | 1 | 0.833 | | |
| | GLY | 0.313 | 0.528 | 0.047 | <0.001 |
| | LEU | 0.801 | 0.976 | 0.05 | <0.001 |
| | PHE | 0.796 | 0.897 | 0.058 | <0.001 |
| | VAL | 0.582 | 0.878 | 0.055 | <0.001 |
| AA2 | ALA | 1.241 | 0.807 | 0.191 | <0.001 |
| | CIT | 1 | 0.543 | | |
| | Fatty Liver | 0.323 | 0.208 | 0.281 | 0.25 |
| | Matsuda Index | -1.61 | -0.367 | 0.529 | 0.002 |
| | MET | 0.828 | 0.519 | 0.168 | <0.001 |
| | OB | 0.36 | 0.273 | 0.222 | 0.104 |
| | ORN | 1.973 | 0.784 | 0.308 | <0.001 |
| | PRO | 1.446 | 0.634 | 0.255 | <0.001 |
| | TYR | 1.553 | 0.883 | 0.229 | <0.001 |
| AC1 | AA2 | 0.372 | 0.475 | 0.093 | <0.001 |
| | AA1 | 0.692 | 0.355 | 0.178 | <0.001 |
| | C2 | 2.432 | 0.82 | 0.35 | <0.001 |
| | C3 | 2.233 | 0.967 | 0.324 | <0.001 |
| | C4 | 1 | 0.556 | | |
| | INFL | 1.49 | 0.326 | 0.6 | 0.013 |
| | Matsuda Index | 0.362 | 0.105 | 0.312 | 0.247 |
| AC2 | AA2 | -0.096 | -0.197 | 0.084 | 0.252 |

| | | | | | |
|---|---|---|---|---|---|
| | AA1 | -0.088 | -0.072 | 0.195 | 0.654 |
| | C10 | 1.26 | 0.834 | 0.136 | <0.001 |
| | C10:1 | 0.603 | 0.711 | 0.074 | <0.001 |
| | C10:2 | 0.318 | 0.31 | 0.091 | <0.001 |
| | C12 | 0.902 | 0.853 | 0.095 | <0.001 |
| | C12:1 | 0.668 | 0.815 | 0.074 | <0.001 |
| | C14 | 0.692 | 0.532 | 0.116 | <0.001 |
| | C14:1 | 0.854 | 0.865 | 0.09 | <0.001 |
| | C14:2 | 1 | 0.699 | | |
| | C5 | 0.584 | 0.478 | 0.106 | <0.001 |
| | C6 | 0.557 | 0.459 | 0.108 | <0.001 |
| | C8 | 1.076 | 0.689 | 0.139 | <0.001 |
| | Fatty Liver | -0.696 | -0.921 | 0.441 | 0.114 |
| | INFL | -0.194 | -0.068 | 0.471 | 0.68 |
| | OB | -0.492 | -0.767 | 0.36 | 0.171 |
| AC3 | AA2 | 0.47 | 0.732 | 0.351 | 0.18 |
| | AA1 | 1.192 | 0.748 | 0.767 | 0.121 |
| | C0 | 0.694 | 0.594 | 0.1 | <0.001 |
| | C16 | 1 | 0.766 | | |
| | C16:1 | 1.048 | 0.261 | 0.35 | 0.003 |
| | C18:1 | 1.28 | 0.869 | 0.124 | <0.001 |
| | C18:2 | 1.156 | 0.665 | 0.149 | <0.001 |
| | Fatty Liver | 3.506 | 3.524 | 1.395 | 0.012 |
| | OB | 2.903 | 3.436 | 1.2 | 0.016 |
| AC4 | AA2 | -0.137 | -0.244 | 0.265 | 0.606 |
| | AA1 | -0.373 | -0.268 | 0.583 | 0.523 |
| | C18 | 1 | 0.654 | | |
| | C18:1OH | 3.779 | 0.273 | 1.308 | 0.004 |
| | Fatty Liver | -2.523 | -2.91 | 1.033 | 0.015 |
| | Matsuda Index | 0.428 | 0.175 | 0.231 | 0.064 |
| | OB | -2.142 | -2.91 | 0.91 | 0.019 |
| Obesity | INFL | 5.245 | 1.184 | 2.842 | 0.065 |
| | Abd_circunf | 0.905 | 0.811 | 0.114 | <0.001 |
| | BMI | 1.26 | 0.922 | 0.146 | <0.001 |
| | FAT | 1 | 0.644 | | |

| | | | | | |
|---|---|---|---|---|---|
| | PCR | 3.669 | 0.907 | 1.262 | 0.004 |
| FATTY LIVER | INFL | -4.918 | -1.307 | 2.53 | 0.052 |
| | ALT | 1 | 0.448 | | |
| | AST | 0.339 | 0.258 | 0.083 | <0.001 |
| | Matsuda Index | -1.048 | -0.371 | 0.311 | <0.001 |
| | NAFLD | 3.405 | 0.752 | 0.673 | <0.001 |
| | PCR | -0.929 | -0.271 | 1.043 | 0.373 |
| INFL | IL-6 | 0.581 | 0.442 | 0.219 | 0.008 |
| | TNFa | 1 | 0.749 | | |
