# Supplementary material for: Family history and obesity in youth, their effect on acylcarnitine/aminoacids metabolomics and non-alcoholic fatty liver disease (NAFLD). Structural equation modeling approach
Source: PLoS One. 2018 Feb 21;13(2):e0193138. doi: 10.1371/journal.pone.0193138 (PMC5821462; doi:10.1371/journal.pone.0193138)
Supplement: S3 Table — Standardized and not standardized β values (β = not standardized estimate, Std β = standardized estimate). BMI = Body mass Index. Abd_circumf = abdominal circumference, FAT = % of Fat. AC1, AC2, AC3 and AC4 = factors grouped for acylcarnitines C2-C18:2. AA1 and AA2 factors grouped for aminoacids. ALA = alanine, CIT = citrulline, Met = methionine, TYR = tyrosine, ORN = ornithine, PRO = proline, ARG = arginine, GLY = glycine, LEU = leucine, PHE = phenylalanine, VAL = valine. CRP = C reactive protein. INFL = inflammatory markers, TNF-a = Tumor necrosis factor alpha, IL-6 = Interleukine 6. USG: liver ultrasound. ALT = Alanine aminotransferase, AST = Aspartate aminotransferase. IndFOB = Second degree family history of obesity. DadFHOB = Parental history of obesity. MomFHOB = Maternal family hitory of Obesity. (PDF) [file pone.0193138.s003.pdf]

S3 Table. SEM Model 3 estimates.

Factors/variables		β	β std	S.E	p-value
Family History	Obesity	0.069	0.223	0.047	0.138
	DadFHOH	0.808	0.548	0.455	0.076
	IndFHOH	1	0.527		
	MomFHOH	0.451	0.274	0.254	0.076
AA1	AC1	-0.065	-0.134	0.091	0.476
	AC2	0.517	0.651	0.135	<0.001
	AC3	0.179	0.302	0.046	<0.001
	ARG	1	0.843		
	GLY	0.312	0.54	0.046	<0.001
	INFL	-1.654	-0.667	0.686	0.016
	LEU	0.798	0.976	0.048	<0.001
	Matsuda Index Index	0.14	0.082	0.344	0.684
	PHE	0.794	0.902	0.055	<0.001
	VAL	0.58	0.883	0.053	<0.001
AA2	AC1	0.778	0.649	0.275	0.005
	AC2	-0.836	-0.427	0.309	0.007
	AC4	0.512	0.278	0.18	0.004
	ALA	1.242	0.819	0.183	<0.001
	CIT	1	0.558		
	INFL	2.973	0.485	1.916	0.121
	Matsuda Index Index	-1.719	-0.407	0.992	0.083
	MET	0.816	0.526	0.16	<0.001
	ORN	1.969	0.794	0.294	<0.001
	PRO	1.434	0.643	0.244	<0.001
	TYR	1.54	0.885	0.218	<0.001
AC1	C2	2.471	0.81	0.364	<0.001
	C3	2.323	0.982	0.344	<0.001
	C4	1	0.543		

	Fatty Liver	-0.027	-0.056	0.042	0.528
	INFL	1.168	0.229	0.654	0.074
	Matsuda Index Index	0.3	0.085	0.401	0.454
AC2	C10	1.264	0.832	0.138	<0.001
	C10:1	0.605	0.71	0.075	<0.001
	C10:2	0.317	0.306	0.092	<0.001
	C12	0.908	0.854	0.097	<0.001
	C12:1	0.671	0.815	0.075	<0.001
	C14	0.694	0.53	0.117	<0.001
	C14:1	0.857	0.864	0.091	<0.001
	C14:2	1	0.695		
	C5	0.593	0.48	0.108	<0.001
	C6	0.557	0.455	0.109	<0.001
	C8	1.075	0.684	0.141	<0.001
	Fatty Liver	-0.437	-1.506	0.199	0.028
	Matsuda Index Index	1.432	0.664	4.097	0.727
AC3	Fatty Liver	1.339	3.451	0.548	0.015
	INFL	1.997	0.478	1.428	0.162
	C16	1	0.753		
	C18:1	1.267	0.848	0.127	<0.001
	C18:2	1.16	0.656	0.154	<0.001
	C16:1	1.016	0.248	0.359	0.005
	Matsuda Index	-4.605	-1.599	12.573	0.714
	C0	0.707	0.594	0.102	<0.001
AC4	Fatty Liver	-0.74	-2.394	0.321	0.021
	C18	1	0.706		
	C18:1OH	3.704	0.287	1.262	0.003
	INFL	-1.522	-0.457	1.157	0.188
	Matsuda Index	2.975	1.296	7.035	0.672
Obesity	AA1	0.7	0.368	0.181	<0.001
	AA2	0.336	0.435	0.087	<0.001

	Abd_circunf	0.885	0.805	0.109	<0.001
	AC2	0.033	0.022	0.14	0.812
	AC3	0.286	0.252	0.09	0.002
	BMI	1.261	0.935	0.141	<0.001
	FAT	1	0.656		
	INFL	-1.554	-0.329	1.542	0.314
	PCR	3.237	0.813	0.829	<0.001
FATTY LIVER	ALT	2.81	0.48	0.637	<0.001
	AST	1	0.289		
	INFL	0.76	0.071	3.612	0.833
	Matsuda Index	0.244	0.033	8.987	0.978
	NAFLD	9.947	0.839	3.276	0.002
	PCR	-1.661	-0.183	1.893	0.38
INFL	IL-6	1	0.824		
	TNFa	0.475	0.395	0.258	0.065
